# The effects of food provisioning on the gut microbiota community and antibiotic resistance genes of Yunnan snub-nosed monkey

**DOI:** 10.3389/fmicb.2024.1361218

**Published:** 2024-03-19

**Authors:** Lei Li, Shan Jing, Yun Tang, Dayong Li, Mingsen Qin

**Affiliations:** ^1^Key Laboratory of Southwest China Wildlife Resources Conservation (Ministry of Education), China West Normal University, Nanchong, China; ^2^School of Electrical Information Engineering, Chengdu Textile College, Chengdu, China

**Keywords:** food provisioning, Yunnan snub-nosed monkey, gut microbiota, antibiotic resistance genes, microbial network

## Abstract

Yunnan snub-nosed monkeys (*Rhinopithecus bieti*) are the highest elevation lived non-human primate, and their survival has been threatened for decades. To promote their population growth, a reserve provides a typical monkey population with supplemental food. However, the influences of this food provisioning on their gut microbiota and antibiotic resistance genes (ARGs) were unknown. Therefore, we investigated the gut microbiota and ARGs of the food-provisioned monkey population compared with another wild foraging population. We found that food provisioning significantly increased the gut microbiota diversity and changed the community composition, particularly increased both the Firmicutes abundance and Firmicutes/Bacteroidetes ratio. Meanwhile, the food provisioning decreased the complex and stable gut microbiota network. KEGG functions were also influenced by food provisioning, with wild foraging monkeys showing higher functions of metabolism and genetic information processing, especially the carbohydrate metabolism, while food-provisioned monkeys exhibited increased environmental information processing, cellular processes, and organismal systems, including valine, leucine, and isoleucine degradation. In addition, food provisioning increased the abundance of ARGs in the gut microbiota, with most increasing the abundance of bacA gene and changing the correlations between specific ARGs and bacterial phyla in each population. Our study highlights that even food provisioning could promote wildlife nutrient intake, and it is necessary to pay attention to the increased ARGs and potential effects on gut microbiota stability and functions for this human conservation measure.

## Introduction

Gut microbiota play an important role in food digestion, absorption, and metabolism of the host, as well as in building the gut barrier and defense system (Lee and Hase, 2014; Zheng et al., 2020). They help maintain the normal functions of the gut and the health of the host (Jandhyala et al., 2015; Rosshart et al., 2017). In this relationship, the host provides a nutrient-rich and stable environment for the gut microbiota (Tsuji et al., 2008) and can influence the composition and functions of gut microbiota through various factors, such as diet, phylogeny, disease, age, and lifestyle (Gerritsen et al., 2011). Among these factors, diet, including its diversity and nutrient intake, is the most important one (Tomova et al., 2019; Cui et al., 2022).

Kilburn et al. (2020) found that a high-fat, high-protein diet increases the abundance of *Bacteroidetes*, and Wu et al. (2011) showed that a high-carbohydrate, high-fiber diet increases the abundance of *Prevotella*. In addition, previous studies have confirmed that diet can influence host health and resistance to disease by influencing gut microbiota (Makki et al., 2018; Catalkaya et al., 2020). All of these studies mainly related to human and rodents, while studies on non-human primates are relatively rare (Nagpal et al., 2018; Cui et al., 2022; Yang et al., 2023). This is important because non-human primates are the closest phylogenetic relatives of humans compared with other animals, making them valuable for understanding microbiota–host interactions and human coevolution (Ley et al., 2008; Yang et al., 2022). Therefore, it is necessary to investigate the impact of diet on the gut microbiota of non-human primates.

The Yunnan snub-nosed monkey (*Rhinopithecus bieti*) is a rare and endemic endangered species in China. It is the highest-distributed non-human primate and lives in the high-latitude forests (3,000–4,400 m) of northwestern Yunnan Province and southeastern Tibet Autonomous Region of China (Xia et al., 2020). Approximately 40 years ago, *R. bieti* was more endangered due to the threat of habitat reduction and fragmentation and hunting (Liu et al., 2009). With the establishment of the Yunnan Baima Snow Mountain National Nature Reserve, the population of *R. bieti* recovered and reached approximately 3,800 individuals (Zhao et al., 2019). To further increase the population and address the food shortage in the wild, the reserve has supplied artificial foods to one representative natural population of *R. bieti*. This supplementary food consists of native lichens, carrots, peanuts, apples, eggs, or pumpkin seeds (Zhu et al., 2016). Artificial food supply contributes greatly to the population expansion of *R. bieti*, but whether there is a negative effect on the health is unknown, especially for the gut microbiota.

Previous studies mainly explored the effect of diet on gut microbiota community composition and diversity, but they have widely ignored the impact on the gut microbiota network (Li et al., 2016; Huang et al., 2021). Microbiota network plays a critical role in shaping the overall functionality of the gut community (Weiss et al., 2016) because the positive and negative correlations within the network reflect interdependence and competitive exclusion, respectively (Wagg et al., 2019). Therefore, confirming the influence of food provisioning on gut microbiota network can help to understand its impact on gut microbiota.

Antibiotic resistance genes (ARGs) can be transmitted horizontally through mobile genetic elements or the food chain in the environment (Ramsamy et al., 2022). Their enrichment within gut microbiota can enhance the antibiotic resistance of pathogenic microbiota, leading to significant difficulty in potential treatment of infections (Allen et al., 2010). In food provisioning, the increased frequency of contact with humans and artificial food may facilitate the transfer of ARGs to gut microbiota (Allen et al., 2010; Hu et al., 2017). However, this potential effect on the abundance and diversity of ARGs has yet to be confirmed.

In this study, we compared two populations of *R. bieti* living in the Yunnan Baima Snow Mountain National Nature Reserve in China. One population foraged naturally, while the other received supplemental food. Both populations lived under similar environmental conditions. The aim of our study was to answer two questions: (1) How does food provisioning affect the gut microbiota community of *R. bieti*, especially the microbiota network? (2) How does the ARGs in the gut change due to food provisioning? Our results will help to evaluate the potential biological risks associated with food provisioning from the perspective of the gut microbiota community and ARGs and provide scientific support for the future conservation of the wild *R. bieti* population.

## Materials and methods

### Study area and food provisioning method

The Baima Snow Mountain National Nature Reserve (98°57′-99°25 E, 27°24′-28°36′ N) is located in Weixi County, Yunnan Province, China. The reserve has an annual average daily temperature of 9.4°C, ranging from a minimum of 2.7°C in January to a maximum of 16.2°C in June. The annual rainfall of the reserve is 1,371 mm, with 70% concentrated between June and October (Li et al., 2023). The typical vegetation is coniferous forest, deciduous broad-leaved forest (2,500–3,600 m), and subalpine fir forest (Zhu et al., 2016). The diet of wild *R. bieti* living in the reserve includes lichens, mature leaves, fruit seeds, young leaves, bamboo shoots, buds, flowers, insects, and fungi (Xia et al., 2022). The wild foraging (WF) population is located in Anyi (99°09′ E, 27°27′ N), and the food-provisioned (FP) population is located in Xianguqing (99°21′ E, 27°39′ N). The FP population consisted of approximately 80 individuals and has been provisioned food since 2009. Every day at 9:00 a.m. and 17:00 p.m., this population receives 10 kg of lichens and 4 kg of other food, including peanuts, apples, pumpkin seeds, and eggs. This supplementary food makes up approximately 40% of FP population daily diet, while the bamboo shoots make up 32% of WF population daily diet, which is significantly higher than that of the FP population (Xia et al., 2022).

### Fecal sample collection

Between March and June, reserve forest rangers used telescopes to locate and observe the monkeys from a distance of approximately 200 meters. Once an individual monkey defecated and moved away, fecal sample was collected. Any adhering plant litter or soil was removed with sterilized tweezers. The collected fecal samples were immediately stored in liquid nitrogen and transported to the laboratory for storage in a −80°C freezer. In total, we collected 92 fecal samples, and 46 samples were collected for each population.

### Enzyme activities and nutrients

Freeze-dried fecal samples (0.1 g) were mixed with 1 mL of ice-cold phosphate buffer (pH 7.5). The mixture was centrifuged at 12,000 r/min for 20 min, and the supernatant was used for enzyme activity and nutrient measurements. Protease (EC 3.4.21) activity was measured using the Folin-phenol method (McDonald and Chen, 1965); amylase (EC 3.2.1.1) activity was measured using the starch-iodine method (Xiao et al., 2006); lipase (EC 3.1.1.3) activity was measured using the polyvinyl alcohol olive oil emulsion hydrolysis method (Andersson, 1980); cellulase (EC 3.2.14) activity was measured using the 3,5-dinitrosalicylic acid method (Breuil and Saddler, 1985). The protein content was measured using the Coomassie brilliant blue dual-wavelength method (Sedmak and Grossberg, 1977); the glucose content was measured using the 3,5-dinitrosalicylic acid method (Borji et al., 2017). A suitable amount of freeze-dried feces was mixed with five times of its weight of sterile water, and then it was used to measure the pH.

### DNA extraction and 16S rRNA sequencing

Total DNA was extracted from 0.25 g fecal samples using the E.Z.N.A.^®^ soil DNA kit (Omega Bio-tek, Norcross, GA, USA), and the extracted DNA was then checked using 1% agarose gel and NanoDrop One Microvolume UV–VIS Spectrophotometer (Thermo Fisher Scientific, MA, USA). PCR amplifications were performed in triplicate to amplify the V3-V4 hypervariable region of the 16S rRNA gene using the primer pair 338F and 806R (Liu et al., 2016). The PCR products were examined and subsequently subjected to high throughput sequencing using Illumina Miseq PE300 platform by a commercial facility (Shanghai Majorbio Bio-Pharm Technology Corporation, Shanghai, China).

The paired-end reads were quality-filtered and spliced using fastp v.0.20.0 (Chen et al., 2018) and FLASH v.1.2.11 (Magoč and Salzberg, 2011). The merged reads were then analyzed on QIIME2 v.2021.4 platform (Bolyen et al., 2019). DADA2 was employed to truncate the reads at 400 bp and perform quality control, read assembly, dereplication, chimera removal, and generation of amplicon sequence variants (ASVs) and the according abundance (Callahan et al., 2016). Singleton ASVs were removed, and the remaining ASVs were taxonomically classified using the feature-classifier classify-sklearn plugin with a confidence score of 0.8 (Bokulich et al., 2018) against the training classifier on Silva 16S rRNA v.138 dataset (Quast et al., 2012). Only bacterial ASVs were retained, and the reads for each sample were resampled to the same depth (3,996 reads).

### Metagenomic sequencing and analysis

Each of 16 fecal samples from the feces of WF and FP monkeys was randomly selected for metagenomic sequencing (Supplementary Table S1). Sequencing was performed on an Illumina NovaSeq PE150 platform (Shanghai Majorbio Bio-Pharm Technology Corporation, Shanghai, China), generating 10G of raw data for each sample. The fastp v.0.20.0 was used to remove the adapters and low-quality reads, which were with a length of <50 bp, or an average quality value of <20, or having N bases (Chen et al., 2018). The reads belonging to *R. bieti* genome (NCBI accession number: GCF_001698545) were removed by BWA v.0.7.9a (Li and Durbin, 2009). The remaining reads were assembled into contigs using MEGAHIT v.1.1.2 (Li et al., 2015), and contigs exceeding 300 bp were used to predict open reading frames (ORFs) with Prodigal (Hyatt et al., 2010). Predicted ORFs with a length of ≥100 bp were retrieved and translated into amino acid sequences. CD-HIT v.4.6.1 (Fu et al., 2012) was employed to construct a non-redundant gene catalog with 90% identity and 90% coverage. The high-quality reads were then aligned to this catalog using SOAP aligner v.2.21 (Li et al., 2008), to calculate the gene abundance with a 95% identity threshold. The KEGG annotation was conducted using Diamond v.0.8.35 (Buchfink et al., 2015) against the Kyoto Encyclopedia of Genes and Genomes database^1^ with an *e*-value cutoff of 1e^−5^. Antibiotic resistance annotation for the non-redundant gene catalog was performed using Diamond v.0.8.35 against the ARDB database^2^ with an *e*-value cutoff of 1e^−5^.

### Statistical analysis

Main statistical analyses were conducted using R v.4.3.1 (R Core Team, 2013). Phylogenetic tree was generated for ASV sequences using Qiime2 align-to-tree-mafft-fasttree command. Rooted and unrooted phylogenetic trees were used to calculate phylogenetic diversity (PD) and nearest taxon index with the *ape* and *picante* packages (Kembel et al., 2010; Paradis and Schliep, 2018). Bray–Curtis similarity among gut microbiota communities was calculated with the *vegan* package (Oksanen et al., 2007) and used for principal coordinate analysis (PCoA). Difference in gut microbiota community structure between WF and FP populations was tested using Adonis permutational multivariate analysis of variance (PERMANOVA) with 9,999 permutations. A group of biomarker taxa, which were most sensitive to the food provisioning treatment, were identified using a Random Forest model with the *randomForest* package (Liaw and Wiener, 2014). Differential ASV abundance between WF and FP gut microbiota was visualized using a Manhattan plot, and the *p*-values for this plot were adjusted using the FDR method (Kaler et al., 2020). The relationships between the microbiota community structure and gut environmental conditions including fecal enzyme activities and nutrients were tested by the Mantel test (Oksanen et al., 2007). To further explore the importance of enzyme activity and gut nutrient in explaining variance of the gut microbiota community structure, variance partition was performed based on the hierarchical partitioning theory using the *rdacca.hp* package (Lai et al., 2022). Gradient forests implemented through the *gradientForest* package were used to explore the individual contributions of gut environmental conditions to the variance of each main ASV taxon (Ellis et al., 2012). This analysis included only the 300 most abundant ASVs in WF or FP, and the overall predictive ability of these predictors was calculated as the average proportion of variance explained by the fitted forest.

Spearman’s correlations were calculated among the 500 most abundant ASVs in each population gut microbiota. Then, the results of Spearman’s correlation with |*r*| > 0.5 and *p* < 0.05 were considered significant and then used to construct the network by Gephi v.0.9.3 (Bastian et al., 2009). Meanwhile, 10,000 random networks, which had the same number of nodes and edges as the real networks, were generated for each real network to evaluate whether the significance of observed correlations was caused by the random incidence (Ju et al., 2014). The significance of differences between the properties of real and random networks was tested by the *Z*-test. To compare the gut network stability of the WF and FP populations, the average degree and natural connectivity were tracked as 0–80% nodes were randomly removed from each network, simulating microbial extinction (Peura et al., 2015). Linear discriminant analysis (LDA) effect size (LEfSe) analysis was used to identify differentially abundant KEGG pathway annotation between the gut microbiota of the WF and FP populations. KEGG pathway annotation biomarkers were detected by LEfSe with an LDA score threshold of >3.0, *p* < 0.05 using the *microeco* package (Liu et al., 2021). Finally, a Pearson correlation analysis was used to calculate the correlations between ARGs and bacterial phyla, and only correlations with |*r*| > 0.4 and *p* < 0.05 were considered significant. PCoA and PERMANOVA were also used to test the differences of compositions of KEGG pathways and ARGs between WF and FP populations.

## Results

### Food provisioning increased gut microbiota diversity and changed their community composition

**Figure 1:**
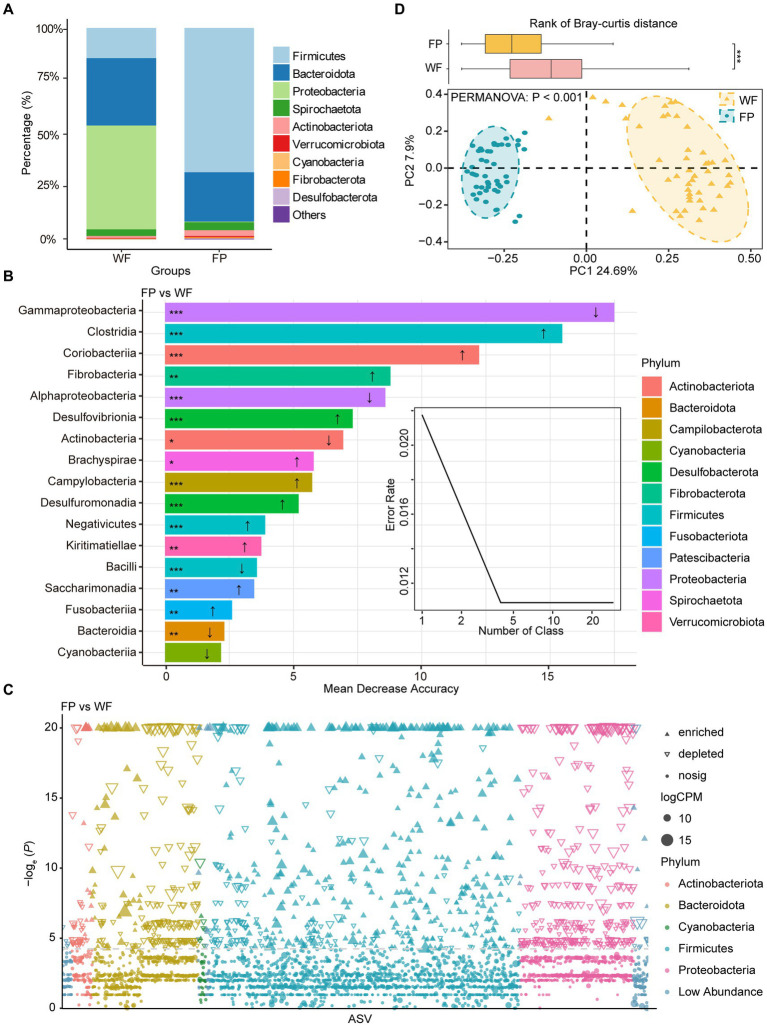
**(A)** Relative abundance of bacterial phyla in wild foraging (WF) and food-provisioned (FP) fecal samples. **(B)** The biomarker taxa listed in descending order of importance to the model accuracy, the up arrow means abundance increasing in FP fecal samples, vice versa. **(C)** Manhattan plots showing enriched or depleted ASVs in WF or FP fecal samples. Threshold = −log_e_ (*p* − value) > 3. **(D)** PCoA of gut microbial communities and ellipses with different colors indicate 95% confidence intervals for each treatment. Boxplot above the PCoA ordination is the comparison of beta diversity of gut microbial communities based on Bray–curtis distance. ***p < 0.001; **p < 0.001; and *p < 0.05.

We found that food provisioning significantly decreased amylase activity and protein concentration (Table 1). It also resulted in a decrease in glucose concentration and an increase in pH, although these changes were not statistically significant (Table 1). From these fecal samples, we got 4,298,438 high-quality sequences, and the average quality score was 38 (Supplementary Figure S1). After resampling to the same depth, 3,973 ASVs were identified from all the fecal samples. The WF population had 2,085 ASVs, while the FP population had 2,363 ASVs, and there were 475 ASVs shared by both populations. Food provisioning significantly enhanced gut microbiota Shannon–wiener diversity, richness, evenness, and phylogenetic diversity and had no significant effect on the nearest taxon index (Table 2). For both WF and FP populations, the gut microbiota phyla were dominated by Firmicutes, Bacteroidetes, Proteobacteria, Spirochaetes, and Actinobacteria (Figure 1A). Notably, Proteobacteria and Firmicutes were the dominant phyla in the WF and FP populations, respectively (Figure 1A). Both their abundance in the dominant population was higher than that observed in the other populations (both *p* < 0.001). Furthermore, we found that the Firmicutes/Bacteroidetes (F/B) was 0.562 in the WF population, which was significantly lower than 5.019 of FP population (Supplementary Figure S2).

**Table 1:** The effect of food provisioning on gut enzyme activities and nutrients.

Physicochemical properties	WF	FP	*p*-value
Protease activity (U/mg)	6.75 ± 0.32	6.84 ± 0.58	0.384
Cellulase activity (U/kg)	7.03 ± 2.77	6.79 ± 1.97	0.637
Amylase activity (U/mg)	3.24 ± 1.78	1.61 ± 1.28	<0.001
Lipase activity (U/g)	0.25 ± 0.13	0.26 ± 0.17	0.903
Protein concentration (μg/μl)	16.45 ± 4.11	12.96 ± 6.92	0.004
Glucose concentration (mg/ml)	5.30 ± 1.21	4.84 ± 1.16	0.066
pH	6.51 ± 0.33	6.63 ± 0.28	0.052

**Table 2:** The effect of food provisioning on gut microbiota diversity and nearest taxon index.

Index	WF	FP	*p*-value
Shannon-wiener	3.57 ± 0.71	4.49 ± 0.48	<0.001
Richness	177.85 ± 57.84	276.46 ± 68.83	<0.001
Evenness	0.69 ± 0.12	0.80 ± 0.06	<0.001
Phylogenetic diversity	14.58 ± 3.08	21.16 ± 2.87	<0.001
Nearest taxon index	3.10 ± 1.10	2.79 ± 1.50	0.253

A Random Forest model was used to identify the most sensitive taxa to food provisioning (Figure 1B). The error curve stabilized with involving the 17 most sensitive classes, which belonged to 12 phyla, and these classes were identified as biomarker taxa (Figure 1B). Among these, the relative abundances of 11 classes, namely, Clostridia, Coriobacteriia, Fibrobacteria, Desulfovibrionia, Brachyspirae, Campylobacteria, Desulfuromonadia, Negativicutes, Kiritimatiellae, Saccharimonadia, and Fusobacteriia, were significantly increased by food provisioning. Conversely, five classes, namely, Gammaproteobacteria, Alphaproteobacteria, Actinobacteria, Bacilli, and Bacteroidia, were significantly reduced by food provisioning (Figure 1B). The Manhattan plot highlighted that food provisioning had a major impact on the ASVs from Bacteroidota, Firmicutes, and Proteobacteria. Food provisioning reduced 179, 126, and 332 ASVs in these phyla, respectively, and increased 39, 395, and 2 ASVs, respectively (Figure 1C). The PCoA showed a distinct clustering and separation of microbiota communities in the gut between WF and FP populations. PERMANOVA confirmed a significant effect of food provisioning on community structure (*p* < 0.001; Figure 1D). Beta-diversity of gut microbiota communities based on Bray–Curtis dissimilarity showed that the WF population had a higher dispersion (Figure 1D).

**Figure 2:**
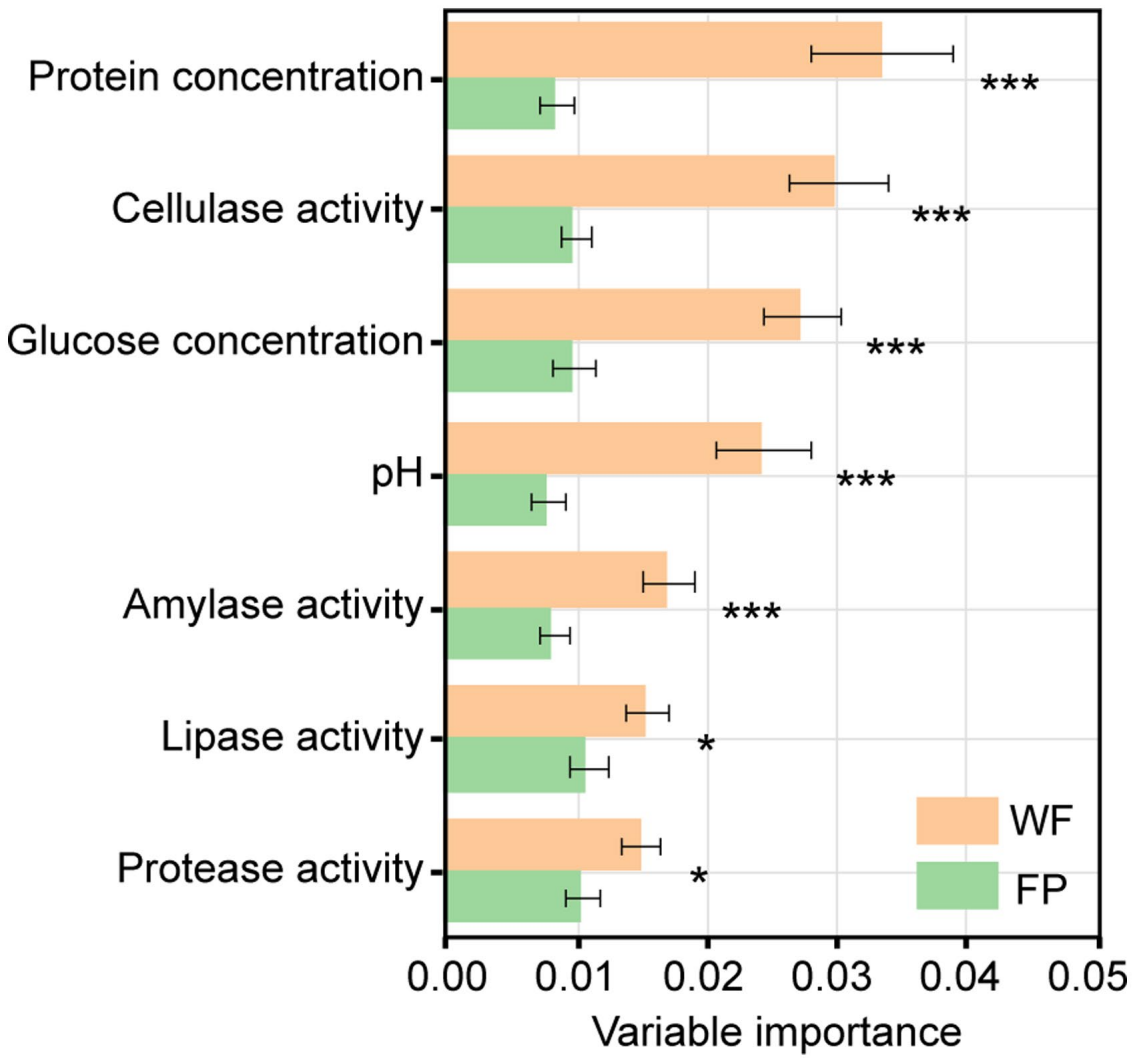
Importance of individual variables of gut enzyme activities and nutrients across models for all predominant ASV taxa. Asterisk means the significant *t*-test of the importance between WF and FP. ***p < 0.001; **p < 0.001; and *p < 0.05.

Mantel tests revealed a significant correlation between the gut microbiota community and a combination of the gut enzyme activities and nutrient contents in the WF population (*r* = 0.334, *p* < 0.001) but not in the FP population (*r* = 0.156, *p* = 0.051; Supplementary Figure S3). For the WF population, of these individual environmental factors, only pH significantly predicted the variance of microbiota community (Supplementary Figure S4). In contrast, no individual factors significantly predicted the variance of gut microbiota community of FP population. For the 300 most abundant ASVs, our gradient forest analysis successfully explained the variance of only 69 ASVs in the WF population and 30 ASVs in the FP population (Supplementary Figure S5). In addition, a t-test showed significantly higher explanation for the WF population (Figure 2). This suggests that the predominant ASVs in the WF population have a tighter correlation with gut environmental conditions compared with the FP population, and it was compatible with the results of the Mantel test.

### Food provisioning decreased gut microbiota network complexity and stability

**Figure 3:**
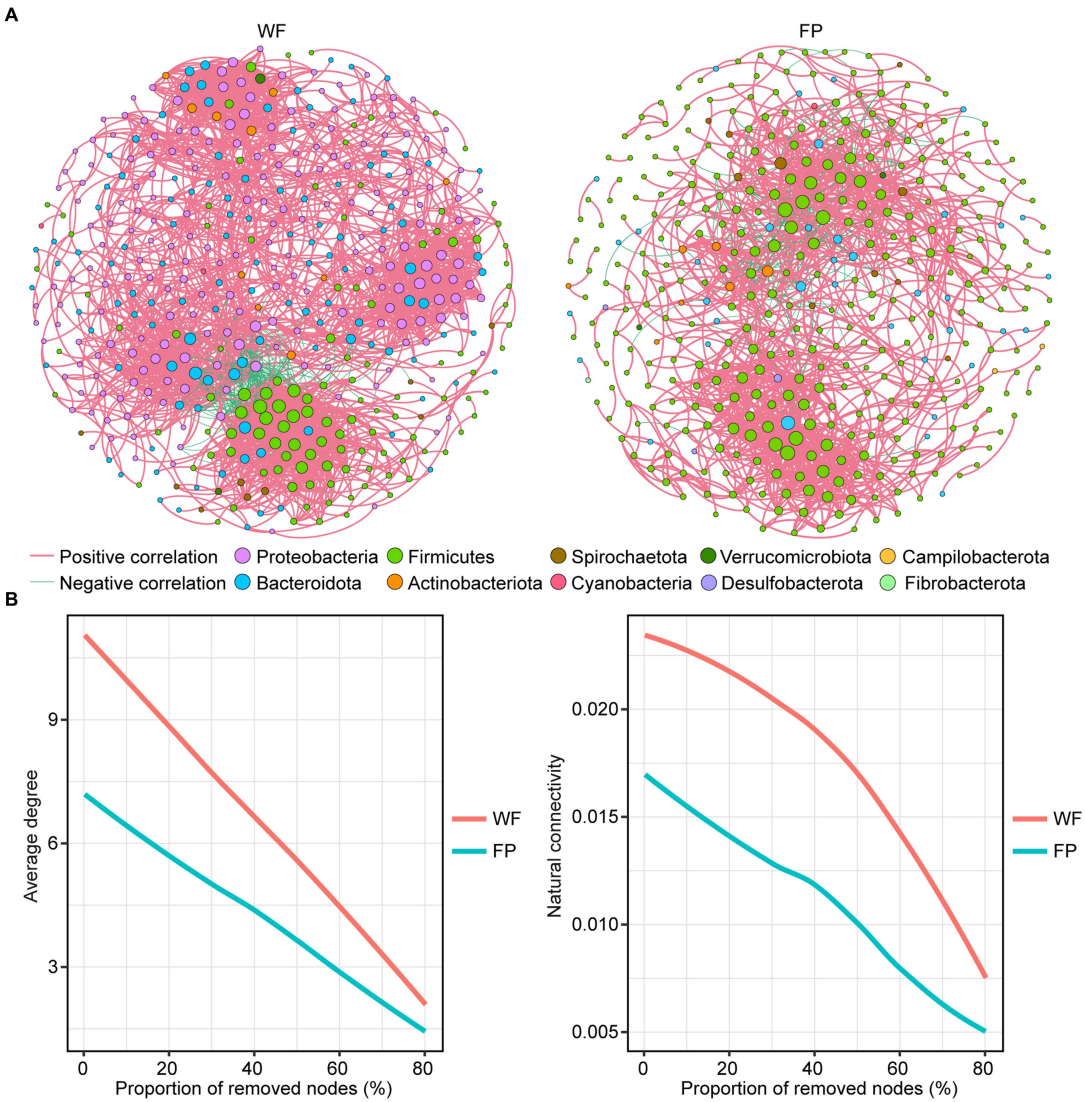
**(A)** Correlation network of gut microbes in WF and FP populations. The node size indicates the relative abundance of the ASV. **(B)** Robustness analysis for gut microbial communities between WF and FP populations by removing the same proportion of nodes.

Comparison of the main properties of real network, including clustering coefficient, modularity, average path length, and network diameter, with those of random network indicated that the real networks were non-random (Table 3). We found that the positive correlation dominated the gut microbiota network of both populations (Table 3). However, compared with the WF population, the gut microbiota network in the FP population had fewer nodes, links, average degree, linkage density, and network diameter (Figure 3A; Table 3). Thus, the WF population had a more complex gut microbiota network. Furthermore, a robustness test showed that the WF population maintained higher average degree and natural connectivity than the FP population as we removed 0–80% of the nodes from the networks (Figure 3B). This demonstrates a greater stability of the gut microbiota network of WF population.

**Table 3:** Characteristics of gut microbiota correlation network.

Parameters	WF	RN of WF	FP	RN of FP	WF − FP
Total nodes	465	465	407	407	58
Total links	2,561	2,561	1,454	1,454	1,107
Positive correlation links	2,389	–	1,296	–	1,093
Negative correlation links	172	–	158	–	14
Average degree	11.015	11.015	7.145	7.145	3.870
Clustering coefficient	0.368^A^	0.023 (±0.002)	0.331^A^	0.018 (±0.002)	0.037
Linkage density	5.508	5.508	3.572	3.572	1.936
Modularity	0.783^A^	0.232 (±0.007)	0.748^A^	0.301 (±0.010)	0.035
Average path length	4.14^A^	2.81 (±0.003)	4.13^A^	3.272 (±0.007)	0.01
Network diameter	13^A^	4.861 (±0.347)	11^A^	6.045 (±0.281)	2

WF, wild foraging population; FP, food-provisioned population; RN, random network. The standard deviation for the property of random network was shown in the bracket. ^A^Significant difference (*p* < 0.001) between the real network and the random network (Z-test).

### Effect of food provisioning on the predicted KEGG pathways

**Figure 4:**
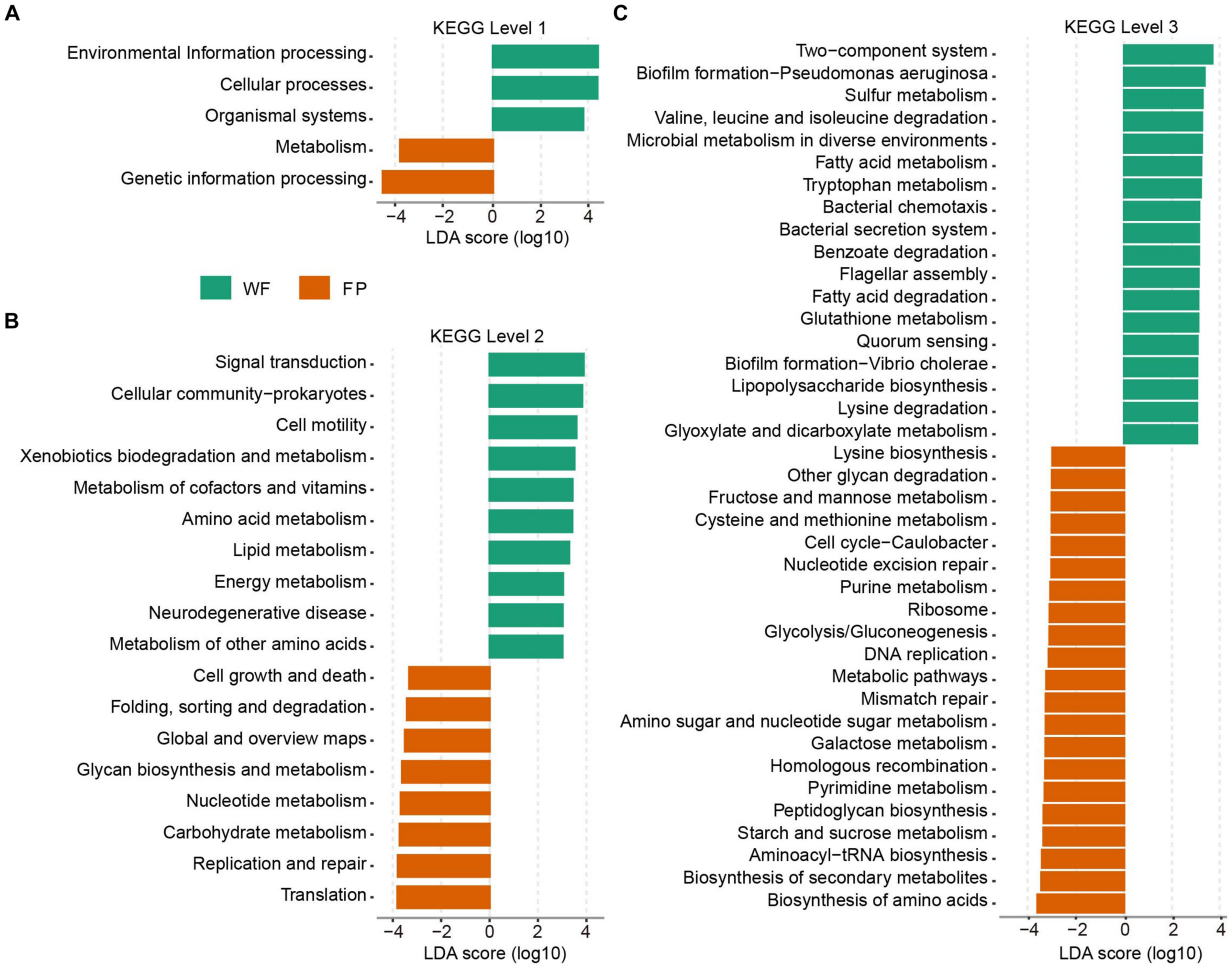
Plots of KEGG pathways comparisons between the WF (green) and FP (orange) populations at levels **(A)** 1, **(B)** 2, and **(C)** 3 analyzed by LEfSe analysis. LDA > 3.0, *p* < 0.05.

KEGG functional analysis revealed that the predicted genes mapped to 6 level 1, 46 level 2, and 446 level 3 pathways. PERMANOVA analysis identified a significant effect of food provision on the overall KEGG pathway composition at all levels (levels 1–3, *p* < 0.001; Supplementary Figure S6). Analyzing KEGG level 1 revealed that the FP population had higher pathways involved in metabolism and genetic information processing, while the WF population had higher pathways involved in environmental information processing, cellular processes, and organismal systems (Figure 4A). At KEGG level 2, the FP population had higher 8 pathways, including the metabolisms of glycan and nucleotide, carbohydrate, and other pathways types such as cell growth and death and global metabolic and overview maps. The WF population had higher 10 pathways, including the metabolisms of cofactor and vitamin, amino acid, lipid, energy, and other amino acids, and other pathways types such as signal transduction, xenobiotic degradation, and metabolism (Figure 4B). At KEGG level 3, the FP population had higher 21 pathways, including the biosynthesis of amino acids, secondary metabolites, aminoacyl-tRNA, peptidoglycan, and lysine, and the metabolisms of starch and sucrose, pyrimidine, galactose, amino sugar, and nucleotide sugar, cysteine and methionine, fructose and mannose, and even the metabolic pathways. The WF population had higher 18 pathways, including the degradation of valine, leucine and isoleucine, benzoate, fatty acid, and lysine and the metabolisms of sulfur, fatty acid, tryptophan, glutathione, glyoxylate, and dicarboxylate (Figure 4C).

### Increased abundance and altered composition of ARGs in the gut microbiota with food provisioning

**Figure 5:**
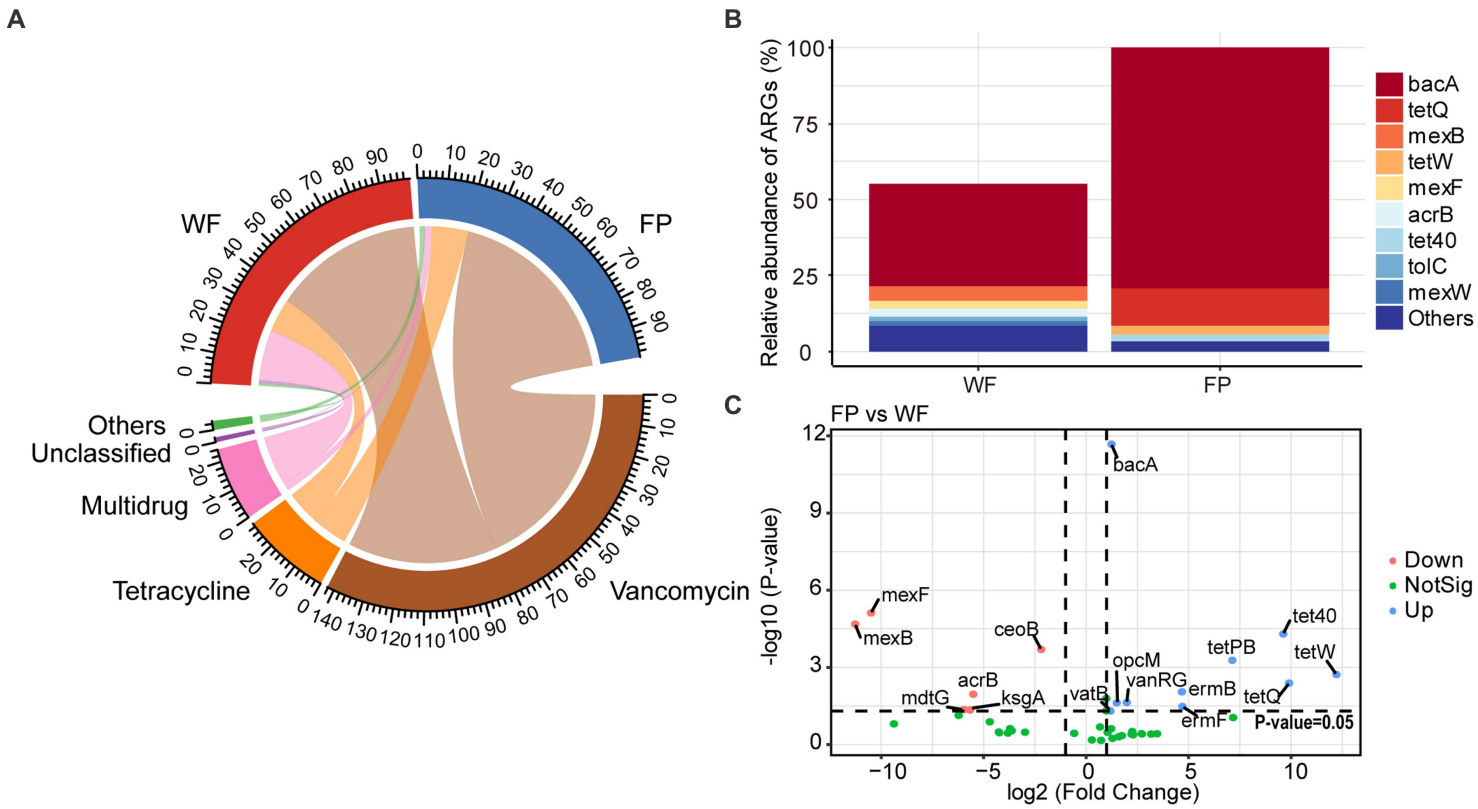
**(A)** Relative abundance of classification of antibiotic resistance genes (ARGs) according to antibiotic resistance type. **(B)** Relative abundance of predominant ARGs comparing to the total abundance of FP population. **(C)** Volcano plot revealed the abundance of differentially ARGs between FP and WF populations.

**Figure 6:**
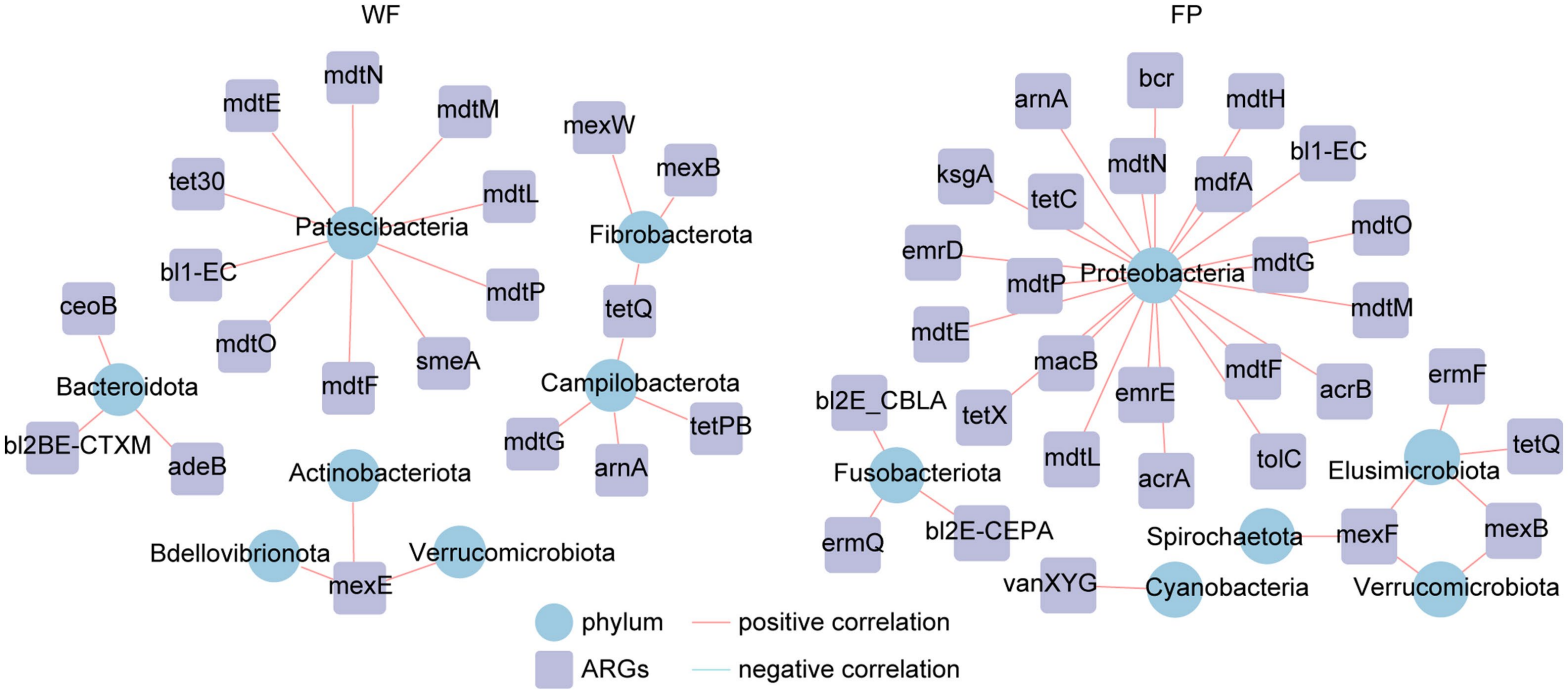
Correlation networks between antibiotic resistance genes (ARGs) and gut bacterial phyla.

Analysis of ARGs in the gut microbiota of the WF and FP populations revealed that vancomycin, tetracycline, and multidrug were the major antibiotic types (Figure 5A). Remarkably, the WF population had a higher abundance of multidrug, while the FP population had higher levels of vancomycin and tetracycline (Figure 5A). Compared with the WF population, food provisioning significantly enhanced the total abundance of ARGs by 81.7% (Figure 5B; Supplementary Figure S7), while food provisioning did change the Shannon diversity but reduced the richness of ARGs (Supplementary Figure S7). ARGs in both WF and FP populations were dominated by the bacA gene (Figure 5B). Food provisioning further increased the abundance of bacA with the greatest increase in the abundance compared with other ARG genes (Figure 5C). In addition, tetW, tetQ, tet40, and tetPB were increased, while mexB, mexF, mdtG, ksgA, and acrB were reduced by food provisioning (Figure 5C). PERMANOVA revealed a change in ARG composition with food provision (Supplementary Figure S8; *p* < 0.001). Our correlation network analysis identified positive correlations between 19 ARGs and 7 phyla taxa in the WF population and between 30 ARGs and 6 phyla taxa in the FP population (Figure 6). Interestingly, Verrucomicrobiota was the only phylum common to both networks (Figure 6). In each population, specific phyla had the most correlated ARGs: Patescibacteria in WF (9 types) and Proteobacteria in FP (22 types).

## Discussion

Previous studies show that diet can influence enzyme activities and nutrient concentrations in the gut (Basolo et al., 2020; Dagar et al., 2023). In this study, food provisioning led to a decrease in gut amylase activity. Similar results were found in studies with nutrient-rich diets (Prakash and Srinivasan, 2012; Saravanan et al., 2014). This could be due to the fact that food provisioning provides sufficient energy, which reduces the body’s need for amylase. In addition, the WF population had higher gut protein concentrations, possibly due to their high intake of protein-rich diet (Xia et al., 2022).

We found that food provisioning increased gut microbiota diversity (Table 2). This could be due to the additional nutrients and carbohydrate substrates provided to the monkeys’ gut, which created more ecological niches for microbiota, ultimately leading to higher diversity (Louis et al., 2007). Food provisioning offered a wider variety of food to the monkey that could also potentially contribute to the increased gut microbiota diversity (Shepherd et al., 2018). However, the increase in ARGs observed in the FP monkeys (Figure 5) suggests another potential impact that increased indirect contact with humans through provisioning of food which could expand the monkeys’ bacterial sources.

Proteobacteria dominated the gut microbiota of WF monkeys and had higher abundance than that of FP monkeys (Figure 1A). Furthermore, Proteobacteria also contained the most sensitive biomarker class (Gammaproteobacteria) response to food provisioning (Figures 1B,C). This is likely due to the high bamboo shoot content in WF diets. Bamboo shoots are rich in unique secondary metabolites such as cyanogenic glycosides (Chongtham et al., 2011). Previous study showed that Proteobacteria had the genes encoding bamboo-degrading enzymes (Sang-A-Gad et al., 2011; Xia et al., 2020), and this is confirmed by the high Proteobacteria abundance in bamboo-eating giant pandas (Zhu et al., 2018). Firmicutes dominated the gut microbiota of FP monkeys, exceeding that of WF monkeys (Figure 1A), and the Firmicutes/Bacteroidetes (F/B) ratio was higher in FP monkeys (Supplementary Figure S2). This raises concerns about potential FP obesity, as the high F/B ratio is a marker of obesity (Crovesy et al., 2020), and we should further monitor the individual obesity of monkeys under food provisioning.

The composition of gut microbiota differed between WF and FP monkeys (Figure 1C). It may be caused by the different diet composition and overall high nutrient intake (Louis et al., 2007). However, according to Cui et al. (2022), a study focusing on another non-human primate, *Macaca mulatta*, suggests that nutrient level exerts a stronger influence on gut microbiota than diet composition. This highlights the need for further exploration of how these factors differentially impact the gut microbiota of *R. bieti*. Interestingly, the FP population displayed lower community dissimilarity within their population compared with WF population (Figure 1C). This result could be explained by the food provisioning that leads to a more consistent diet among individuals, thereby reducing variation in gut microbiota communities (Li et al., 2016). Notably, both Mantel test and gradient forest analysis revealed that the gut microbiota community of the WF population exhibited a stronger correlation with their gut environment. According to the nutrient niche theory, we hypothesize that the increased nutrient intake from food provisioning could make the gut environment more conducive to the growth of gut microbiota, which enhances the resistance of the microbiota community to the changes in the gut environment. This seems to reduce the linkage between the gut environmental condition and gut microbiota community composition (Freter et al., 1983), while this observation figures out that the mechanism behind this result needs further investigation.

The analysis of gut microbiota networks revealed that the positive correlation dominated both populations, suggesting that microbiota growth in their gut mainly relies on collaboration (Layeghifard et al., 2017). Further comparison of the networks revealed that the gut of WF population had a more complex microbiota network (Figure 3A; Table 3). This is mainly because most microbiota require nutrient exchange to grow normally, and when their overall nutrient intake is lower, as in the case of the WF population, their interdependence will increase, which promotes the complexity of their networks (Layeghifard et al., 2017; Zengler and Zaramela, 2018). The network robustness test conducted in our study showed that the gut microbiota network degree and natural connectivity of the WF population after continuous removal of ASVs were always higher than those of the FP population (Figure 3B). This suggests that WF monkeys exhibit greater resilience and stability in their gut microbiota communities (Coyte et al., 2015).

The KEGG pathway analysis showed that food provisioning significantly changed the composition of gut KEGG functions (Supplementary Figure S6), enriching the pathway-related metabolism and genetic information processing (Figure 4A). This is due to the fact that the FP population had higher nutrient intake. In this condition, higher nutrient provides gut microbiota with more raw materials to build their cellular components and enzymes, which needs more gene expression and protein synthesis (Okie et al., 2020). Furthermore, higher nutrient could also enhance to build the pathways for efficient and diverse metabolic processes. However, this metabolic enrichment was not evenly distributed. The KEGG level 2 analysis revealed this enrichment on metabolism pathways that were related to global metabolic and overview maps and metabolisms of carbohydrate, glycan, and nucleotide, while pathways related to xenobiotic degradation and metabolism and metabolisms of cofactor, vitamin, amino acid, lipid, energy, and other amino acids were all depleted. Our food provisioning, including carbohydrate-rich peanut and pumpkin seeds, could trigger the activity of carbohydrate metabolism (Li et al., 2017). Conversely, the low nutrient intake of the WF population forced them to upregulate the pathways involved in breaking down and utilization of other substrates such as xenobiotics, cofactors, vitamins, amino acids, lipids, and energy-generating molecules to meet the energy needs for gut microbiota. Drilling down to KEGG level 3, we observed enhanced degradation pathways in the WF population, specifically for benzoate, valine, leucine, isoleucine, lysine, and fatty acids (Figure 4C). We hypothesized that the food sources of WF population contained more plant toxins such as alkaloids, which potentially induced higher microbiota detoxification efforts, including geraniol breakdown (Chen and Viljoen, 2010). In addition, the higher protein content in WF diets likely provided them with a rich amino acid pool, increasing the degradation of valine, leucine, isoleucine, and lysine (Madsen et al., 2017).

This study confirmed that the food provisioning increased the abundance of ARGs in the gut of *R. bieti* (Figure 5B). This may be because both human contact and artificial food intake during food provisioning could increase the horizontal transformation of ARGs to the gut (Allen et al., 2010; Hu et al., 2017). Another possible reason might be due to the dietary influence as Yan et al. (2022) found that high-fat/low-fiber diet could also increase both abundance and diversity of ARGs of the gut of cynomolgus monkeys (*Macaca fascicularis*). However, our food provisioning had no effect on the Shannon diversity and even reduced the richness of ARGs (Supplementary Figure S7). While the bacA was the most abundant and increased ARGs in our study (Figure 5B), this ARG type abundance was not changed by the high-fat diet in the study of Yan et al. (2022). Thus, we speculated that the horizontal transformation was the main mechanism to enrich the abundance of ARGs in the gut of *R. bieti*, but both possible mechanisms should be investigated in the future. In this study, bacA was dominated in both WF and FP populations among the ARGs. This result is also found from human (Feng et al., 2018; Li et al., 2021) and other animals (Thomas et al., 2021; Zhang et al., 2022). Meanwhile, the most increased ARGs by food provisioning were also the bacA (Figure 5B), which might be due to its high abundance in human guts (Feng et al., 2018; Li et al., 2021), facilitating its more and effortless transfer to FP monkeys. Another finding is that the abundance of tetracycline ARGs, including tetPB, tet40, tetW, and tetQ, was increased by food provisioning (Figure 5C). This could be partly explained by the result about the suppression of gut protein on tetracycline ARGs, which was observed by Zhang et al. (2016). As the FP population had lower gut protein concentration compared with the WF population, this factor might contribute to the increased abundance of these specific ARGs. Our analysis of ARG correlation networks with gut bacterial phyla revealed that food provisioning could alter the correlations between ARGs and specific bacterial phyla (Figure 6). Notably, Patescibacteria had the most correlations with ARGs in the WF population, while Proteobacteria had the highest correlations in the FP population, with a higher number of connections than the WF population. Both phyla have been previously implicated in harboring ARGs (Chen et al., 2017; Kim et al., 2023). We hypothesized that the richer nutritional environment in the FP population might stimulate ARG transfer to Proteobacteria, but further research was needed to unravel the underlying mechanisms.

## Conclusion

Our study demonstrated the complex impact of food provisioning on the gut microbiota of Yunnan snub-nosed monkeys. We observed that while food provisioning increases the diversity of gut microbiota, it also changed the gut microbiota community composition, leading to greater homogeneity of community composition among individuals. The network analysis revealed that food provisioning reduced the complexity and stability of the gut microbiota network, potentially weakening its resilience to disturbance. Furthermore, metagenomic sequencing revealed that food provisioning significantly increases the abundance of ARGs in the gut, potentially raising the risk of drug resistance in this wildlife. These findings underscored the necessity of a comprehensive approach when considering food provisioning for wildlife. While increasing diversity may seem beneficial (Fan and Pedersen, 2021), it was crucial to simultaneously monitor the potential drawbacks, including altered community structure, changed network stability, and increased ARGs, to truly ensure the benefits of this conservation practice.

## Data availability statement

The datasets presented in this study can be found in online repositories. The sequences data is deposited in National Microbiology Data Center (NMDC) with accession number NMDC10018711. (https://nmdc.cn/resource/genomics/project/detail/NMDC10018711).

## Author contributions

LL: Conceptualization, Data curation, Formal analysis, Methodology, Software, Writing – original draft, Writing – review & editing. SJ: Conceptualization, Investigation, Methodology, Software, Validation, Writing – review & editing. YT: Investigation, Project administration, Resources, Writing – review & editing. DL: Data curation, Investigation, Resources, Supervision, Writing – review & editing. MQ: Conceptualization, Data curation, Software, Supervision, Writing – review & editing.

## Footnotes

^1^ http://www.genome.jp/keeg/

^2^ http://ardb.cbcb.umd.edu/
