# Supplementary material for: The effects of food provisioning on the gut microbiota community and antibiotic resistance genes of Yunnan snub-nosed monkey
Source: Front Microbiol. 2024 Mar 19;15:1361218. doi: 10.3389/fmicb.2024.1361218 (PMC10985317; doi:10.3389/fmicb.2024.1361218)
Supplement: Supplementary file 1 [file Table_1.DOCX]

Table S1 The samples used to 16S rRNA and metagenomics highthroughput Sequencing

| Wild foraging | | | | | |  | Food-provisioned | | | | | |
| --- | --- | --- | --- | --- | --- | --- | --- | --- | --- | --- | --- | --- |
| Sample | 16S rRNA | MAGs | Sample | 16S rRNA | MAGs |  | Sample | 16S rRNA | MAGs | Sample | 16S rRNA | MAGs |
| A_1 | * | * | A_24 | * |  |  | X_1 | * | * | X_24 | * |  |
| A_2 | * | * | A_25 | * |  |  | X_2 | * | * | X_25 | * |  |
| A_3 | * | * | A_26 | * |  |  | X_3 | * |  | X_26 | * | * |
| A_4 | * | * | A_27 | * |  |  | X_4 | * | * | X_27 | * | * |
| A_5 | * | * | A_28 | * |  |  | X_5 | * |  | X_28 | * |  |
| A_6 | * | * | A_29 | * |  |  | X_6 | * |  | X_29 | * |  |
| A_7 | * | * | A_30 | * |  |  | X_7 | * | * | X_30 | * | * |
| A_8 | * | * | A_31 | * |  |  | X_8 | * | * | X_31 | * |  |
| A_9 | * | * | A_32 | * |  |  | X_9 | * |  | X_32 | * | * |
| A_10 | * | * | A_33 | * |  |  | X_10 | * |  | X_33 | * |  |
| A_11 | * | * | A_34 | * |  |  | X_11 | * |  | X_34 | * | * |
| A_12 | * | * | A_35 | * |  |  | X_12 | * |  | X_35 | * |  |
| A_13 | * | * | A_36 | * |  |  | X_13 | * |  | X_36 | * |  |
| A_14 | * | * | A_37 | * |  |  | X_14 | * |  | X_37 | * |  |
| A_15 | * | * | A_38 | * |  |  | X_15 | * |  | X_38 | * |  |
| A_16 | * | * | A_39 | * |  |  | X_16 | * |  | X_39 | * | * |
| A_17 | * |  | A_40 | * |  |  | X_17 | * |  | X_40 | * |  |
| A_18 | * |  | A_41 | * |  |  | X_18 | * | * | X_41 | * | * |
| A_19 | * |  | A_42 | * |  |  | X_19 | * |  | X_42 | * |  |
| A_20 | * |  | A_43 | * |  |  | X_20 | * | * | X_43 | * | * |
| A_21 | * |  | A_44 | * |  |  | X_21 | * |  | X_44 | * |  |
| A_22 | * |  | A_45 | * |  |  | X_22 | * |  | X_45 | * | * |
| A_23 | * |  | A_46 | * |  |  | X_23 | * |  | X_46 | * |  |

Figure S1 The sequences quality of 16S rRNA


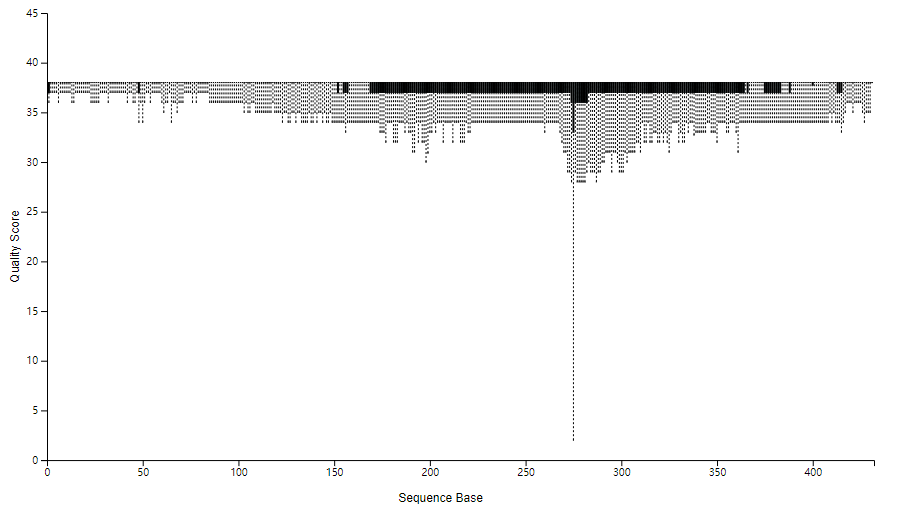


Figure S2 The comparison of gut microbial Firmicutes/Bacteroidetes (F/B) between WF and FP populations. Significance levels are indicated by asterisks: ****P* < 0.001.


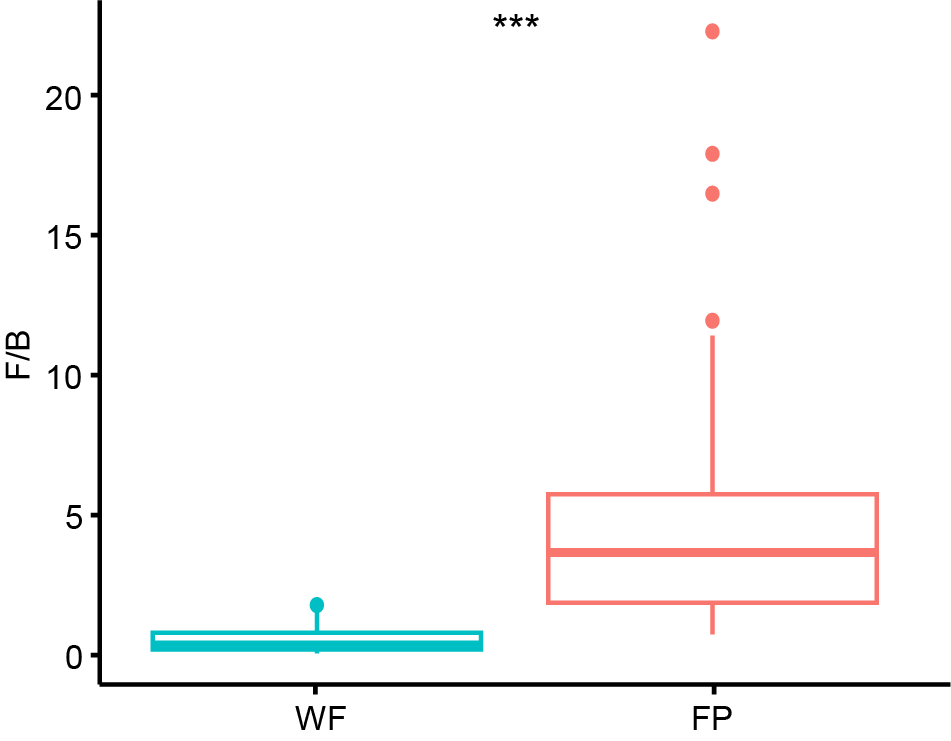


Figure S3 Mantel test of the relationship between soil microbiota community dissimilarity and gut environmental condition distance. The lines denote the ordinary least-squares linear regression in WF and FP populations. Statistics are derived from Mantel test analysis with permutations=10000.


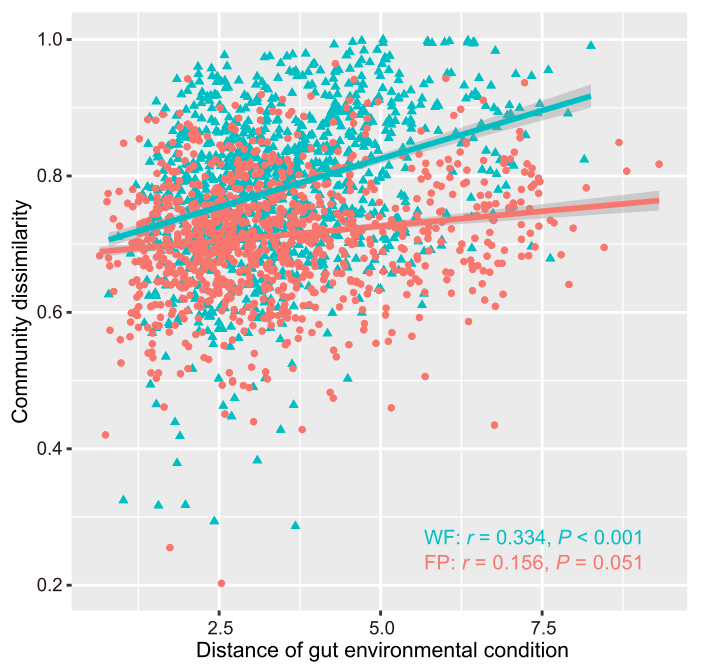


Figure S4 The variance of gut microbial community explained by gut enzyme activities and nutrients using variation partition analysis (VPA). Predictor with a negative value was removed for the last variation partition. Significance levels are indicated by asterisks: ***P < 0.001; **P < 0.001; *P < 0.05.


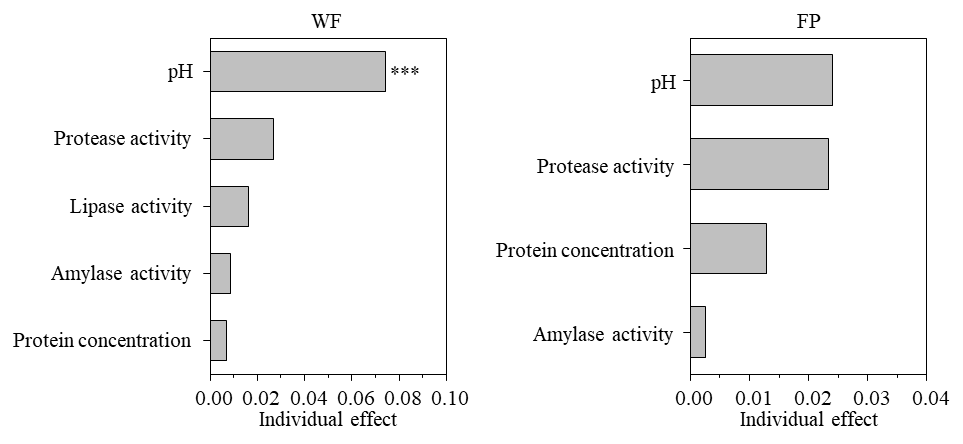


Figure S5 Gut environmental variables explaining the most frequent microbiota taxa of WF and FP populations. Random forest model performance for taxa where out-of-bag *R*^2^ > 0.


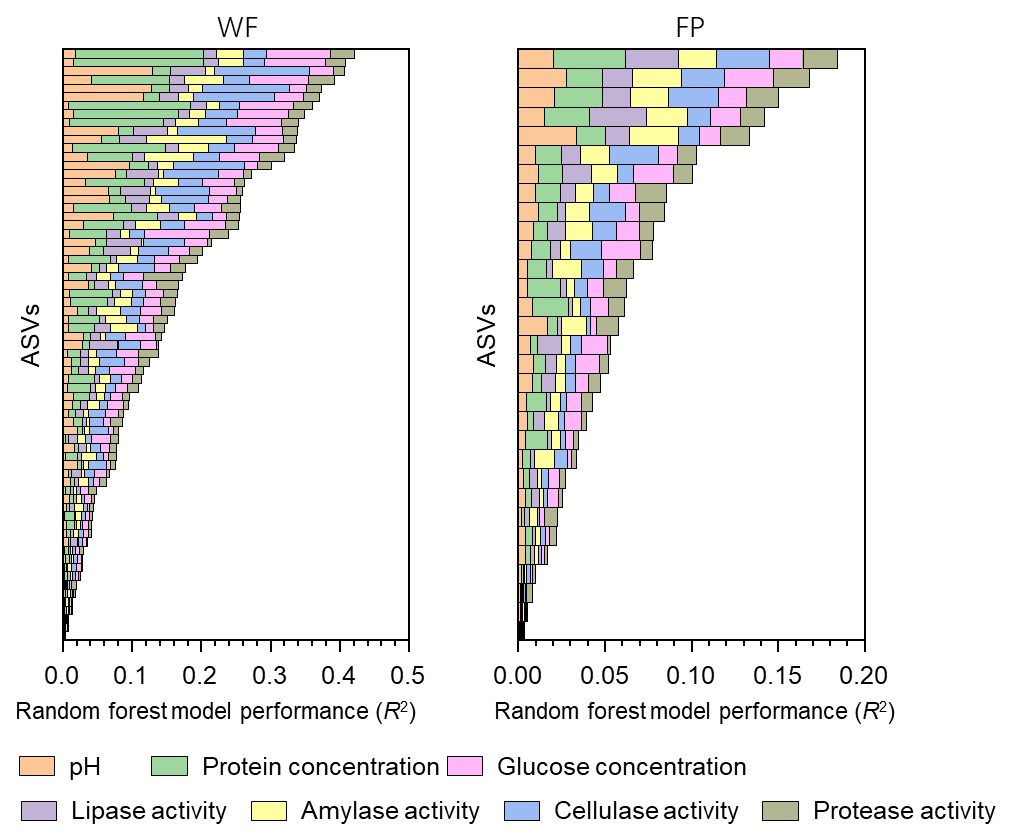


Figure S6 Principal Coordinates Analysis (PCoA) plot for the KEGG pathway annotations and PERMANOVA test the difference of ARGs composition between WF and FP populations.


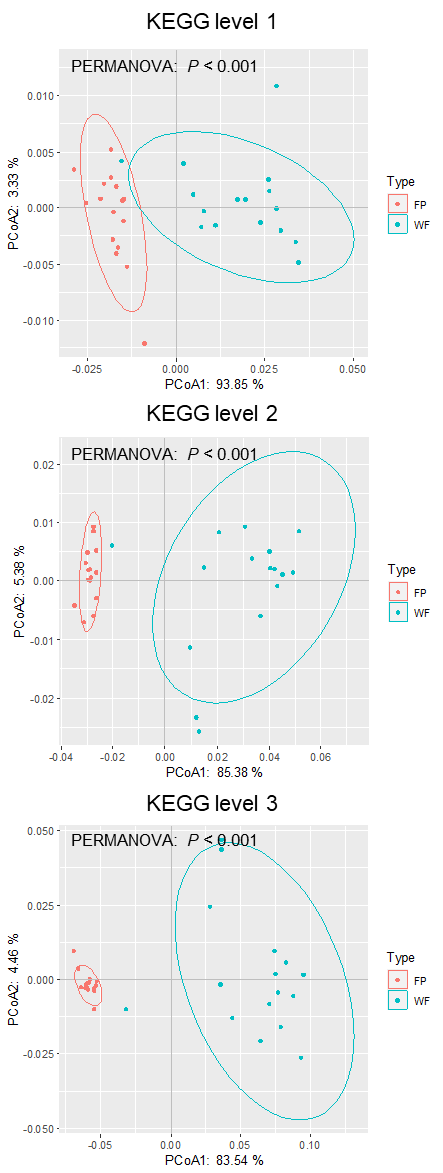


Figure S7 The difference of ARGs Shannon-wiener, richness, and abundance between WF and FP populations.

Figure S8 Principal Coordinates Analysis (PCoA) plot for the ARGs and PERMANOVA test the difference of ARGs composition between WF and FP populations.
